# Barriers to Integration of Primary Care into Emergency Care: Experiences in Germany

**DOI:** 10.5334/ijic.5442

**Published:** 2021-04-26

**Authors:** Andrew Dickinson, Stefanie Joos

**Affiliations:** 1 Universitätsklinikum Tübingen, Institut für Allgemeinmedizin und Interprofessionelle Versorgung, Osianderstraße 5, 72076, DE; 2 Institut für Allgemeinmedizin und Interprofessionelle Versorgung UNIVERSITÄTSKLINIKUM TÜBINGEN

**Keywords:** Words: emergency care systems, primary care practitioner integration, triage, normative integration, integrated care

## Abstract

**Introduction:**

In response to emergency department over-crowding primary care practitioners (PCPs) have been incorporated into care pathways to provide integrated care. We consider why a pilot project of PCP-led streaming in a German emergency department failed, the challenges encountered transplanting models between differing systems and cultures, and if the concept constitutes integrated care.

**Theory and Methods:**

The original design was a mixed methods data gather around PCP-streaming of non-urgent self-referrers in an emergency department.

**Results:**

The demand for the PCP-streaming was low, which was at odds with pre-study estimates. The study was stopped prematurely without adequate data; this is an opinion-based article.

**Discussion:**

A fundamental of emergency care is a central emergency department. An emergency department can be the fulcrum from which urgent inter-disciplinary hospital care is initiated and coordinated. Objective triage is fundamental to this and regional healthcare planning. With such fundamentals in place, PCP integration has the potential to facilitate and provide integrated care. Relevant elements of the Rainbow Model of Integrated Care frame the discussion.

**Conclusion:**

The key element deficient in each barrier to our project, yet present in successful studies, was normative integration.

## Introduction

### Background

#### Overcrowding

There has been a steady increase of patients in emergency departments across nearly all of the Organization for Economic Cooperation and Development [1], which causes ‘overcrowding’ and puts strain on health systems. Germany has seen a doubling of patients in the emergency department to 25 million per year during the period 2005 to 2015 [2]. The causes of this increase are complex, but a significant proportion of these patients are non-urgent and attend without previous contact with a primary care practitioner (PCP) [3].

Part of the response in many healthcare systems has been to incorporate PCPs into emergency care pathways. This has been tried in numerous forms including primary care services setup alongside and in parallel to the emergency department (e.g. PCP co-operatives or Urgent Care Centres), with the hope of alleviating some of the non-emergency-patient demand, and with some limited success [4,5]. Other models have tried placing PCPs *within* the emergency department. These have included: PCPs at the front of the department as triage providers (i.e. risk-stratification and screening of emergency department patients) with some scope for treatment or sign-posting; PCPs seeing and treating patients post-triage who have been streamed into a non-urgent category; PCPs fully immersed within the emergency department as undifferentiated emergency department doctors [6]. Placing of a PCP within the triage process itself is topical in the UK given the political direction for it to be implemented nationwide [7,8] but the evidence of the benefit is not conclusive and of poor quality [5,6,9]. Indeed, this can be said of the overall evidence for PCPs in the emergency department. A recent Cochrane Review of PCPs providing non-urgent care in emergency departments identified four trials (11,463 patients) conducted in Ireland, the UK and Australia. The studies provided very-low certainty and inconsistent results and were deemed insufficient to draw conclusions for practice or policy [10]. This study was designed incorporating recommendations from literature, particularly those from the Cochrane Review, which pre-dated our study [6]. Our aim was to fill the gap in evidence and explore the efficacy of this intervention through a mixed methods feasibility study, which was to be the pre-cursor to a large trial. The hope too was that the pilot would help alleviate the perceived demand from ‘self-referrers’ [11].

#### Integrated Care?

The placement of PCPs within emergency care is often (and perhaps too quickly) referred to as integration. Such changes are typically an organisational effort to enable ‘integration’ but does it manifest as integrated care and what is integrated care?

Kodner was co-author to a comprehensive early definition in 2002 [12], but by 2009 he struck a more exasperated tone acknowledging the breadth of usage, “Integrated care as a concept is an imprecise hodgepodge. Its meanings are as diverse as the numerous actors involved” [13]. However, in the same paper he develops a more distilled definition, “It is a multi-level, multi-modal, demand-driven and patient-centred strategy designed to address complex and costly health needs by achieving better coordination of services across the entire care continuum.”

### Study Context

Tübingen is a historic university town within Baden Württemberg in southern Germany. Its 1900-bed hospital complex is a tertiary referral centre for a vast region within Baden Württemberg and includes 5 separate, sub-specialist ‘Notaufnahmen’ (NA), which approximately translates to ‘emergency department’ (see Discussion). Four of the NA are within The University of Tübingen’s Teaching Hospital (UKT) and the other, the trauma NA, is part of another healthcare provider called Berufsgenossenschaftliche (BG) Klinik. Tübingen lacks a centralised emergency department.

The NAs in Tübingen have experienced an increase in patient throughput. Since 2010 the yearly number of patients in the medical NA has increased on average by 200 per year rising from 8,281 in 2010 to 10,023 in 2015. The proportion of ‘self-referrers’ was estimated to be between 40 and 60% in an internal audit. Since 2016, and in part-response to such demand, there has been a parallel hospital-based PCP Urgent Care service (the Hausärztliche Notdienst) but it is limited to an out-of-hours service. This pilot project was intended to establish the need for a more extensive daily service. The aim was to implement an integrated PCP within the NA to see the non-urgent patients post-streaming and evaluate the impact on pre-determined outcomes (***Table 1***).

**Table 1:** Intended Outcomes.


PRIMARY OUTCOMES	SECONDARY OUTCOMES

Time from presentation to treatment Duration within department Admitted versus discharged	Investigations Therapy Further specialistreferral Onward care Subsequent use of Notaufnahme or Primary Care Cost-effectiveness Adverse events


The PCP for the study was recruited following advertising for academic PCPs in the medical journal of Germany, The Ärzteblatt. The UKT’s Department for General Practice and Multi-Disciplinary Care led the project in conjunction with The Internal Medicine Department I of UKT, which administrated the medical NA.

To gain insight, the study PCP visited the emergency departments of Hannover and Hamburg, which successfully implemented similar PCP integration projects [14,15], and Basel Universitätsspital’s emergency department, which has a well-developed triage and streaming service [16]. These insights were to augment the study PCP’s prior experience of general practice, emergency medicine and pre-hospital emergency medicine in civilian and military settings [17,18].

### Problem statement

This article draws on the experiences of a PCP from the UK establishing a pilot project of PCP-led streaming in an emergency department within Germany. It is an opinion piece, which considers why the project failed, the challenges encountered transplanting models between differing healthcare systems and cultures, and whether the concept truly constitutes integrated care.

## Study Design

**Figure 1:**
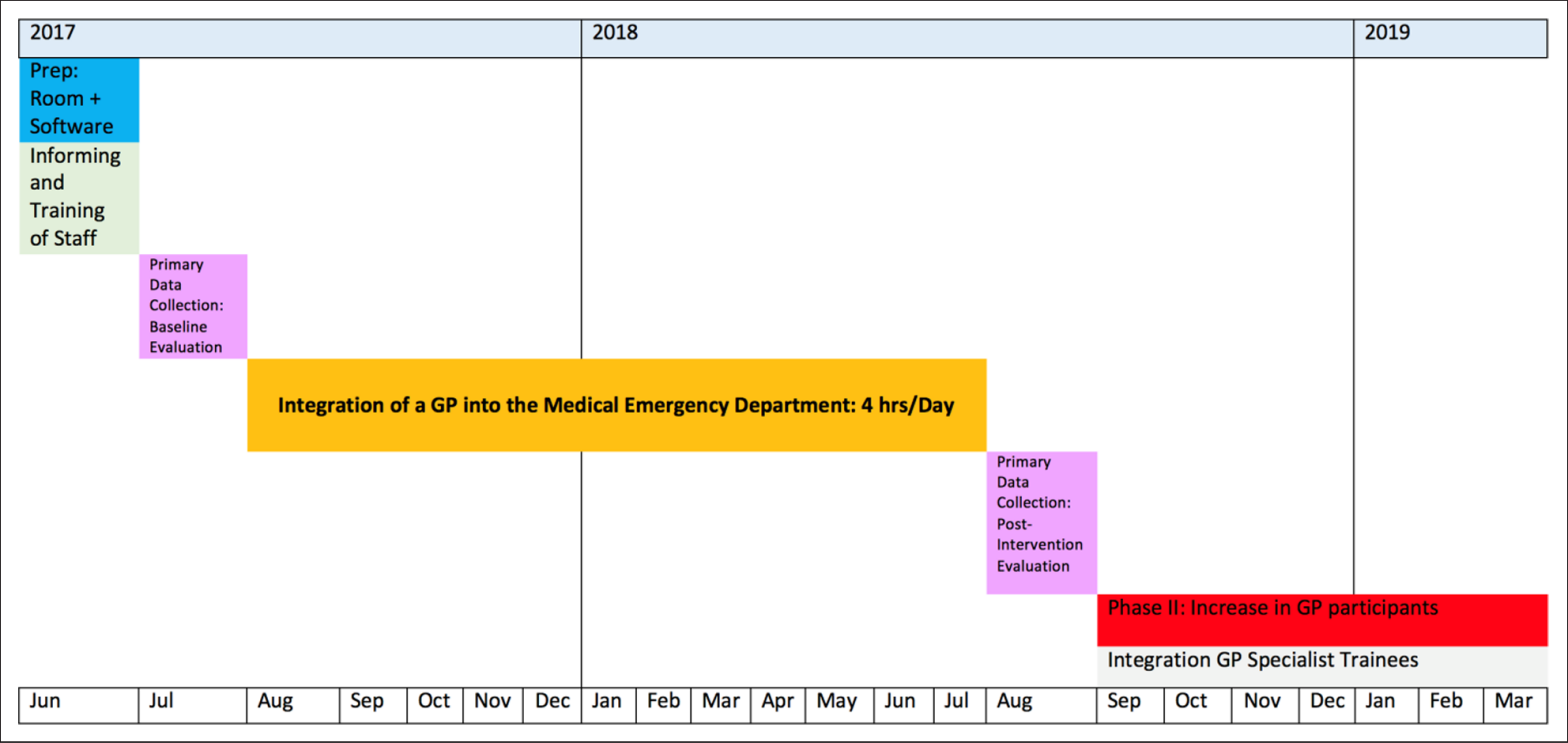
Project Plan.

The pilot project was originally designed with a stepwise implementation (***Figure 1***) and a mixed-methods data gathering plan (***Tables 1*** and ***2***). Ethical approval for the project and its data collation was awarded by the UKT’s ethics committee; project number 329/2017BO2.

**Table 2:** Intended Mixed-Method Data Collection.


QUANTITATIVE DATA			

CLINICAL LEVEL			COMMENTS

Patient Questionnaires	Pre- and Post-Intervention (i.e. PCP-Streaming)	*Pre-consultation*: demographic data and circumstances of attendance	*The questionnaire was designed using the Hamburg study’s questionnaire as a template [15] and augmented as per the Tübingen context and study focus*.

		*Post-treatment*: patient’s experience and satisfaction, care received/ recommended	

**ORGANISATIONAL LEVEL**			

Hospital Clinical Software	Pseudo-anonymised data	Patient journey, resource use and cost of care (to the public body)*	*Outcome was acknowledged to be a likely increase in resource use and cost of care because many self-referrers were previously sign-posted to alternative healthcare access points (e.g. outpatient clinic, primary care practice)*.

**QUALITATIVE DATA**			

Interviews with ED staff	Pre- and Post-Intervention (i.e. PCP-Streaming)	Experiences pre- and post-intervention	*The interviews were planned to take place at the 6-month point, however the project did not reach this stage*.

First person experiences of study PCP			


* We were to reassess and focus on cost comparisons in the broader roll out of the project in the second phase, but the project was terminated early. A recent meta-analysis serves as a useful guide in the cost analysis of integrated care projects [19].

For one month prior to the project, the study PCP was present within the department during the project hours to establish an understanding of the department and inform colleagues of the proposed pilot project. The communication included face to face, posters, emails sent to all personnel linked to the department, and finally a brief introductory lecture to the medical department. The project was not advertised to the public to avoid altering healthcare-seeking behaviours.

The first phase of the project was funded for a 4-hour PCP shift during the working week. The timings were chosen based on medical NA data, which indicated that self-referrers (albeit with different criteria; see Results) peaked between these hours; the pre-study audit data estimated between 11 and 17 self-referrers per day, most of whom attending within the morning 4-hour PCP shift.

The original intention was to collate data pre- and post-intervention. The PCP-streaming was to be in place for 12 months, before and after which questionnaires were to be completed, admission software data analysed, and further qualitative reflections gathered in the Post-Intervention Evaluation period. If the initial results of the pilot were to suggest a benefit from the intervention, then the plan was to increase the number of PCPs and expand the cover over the day of PCP-streaming. However, the project was stopped after 3 months and the intended data largely uncollated.

**Figure 2:**
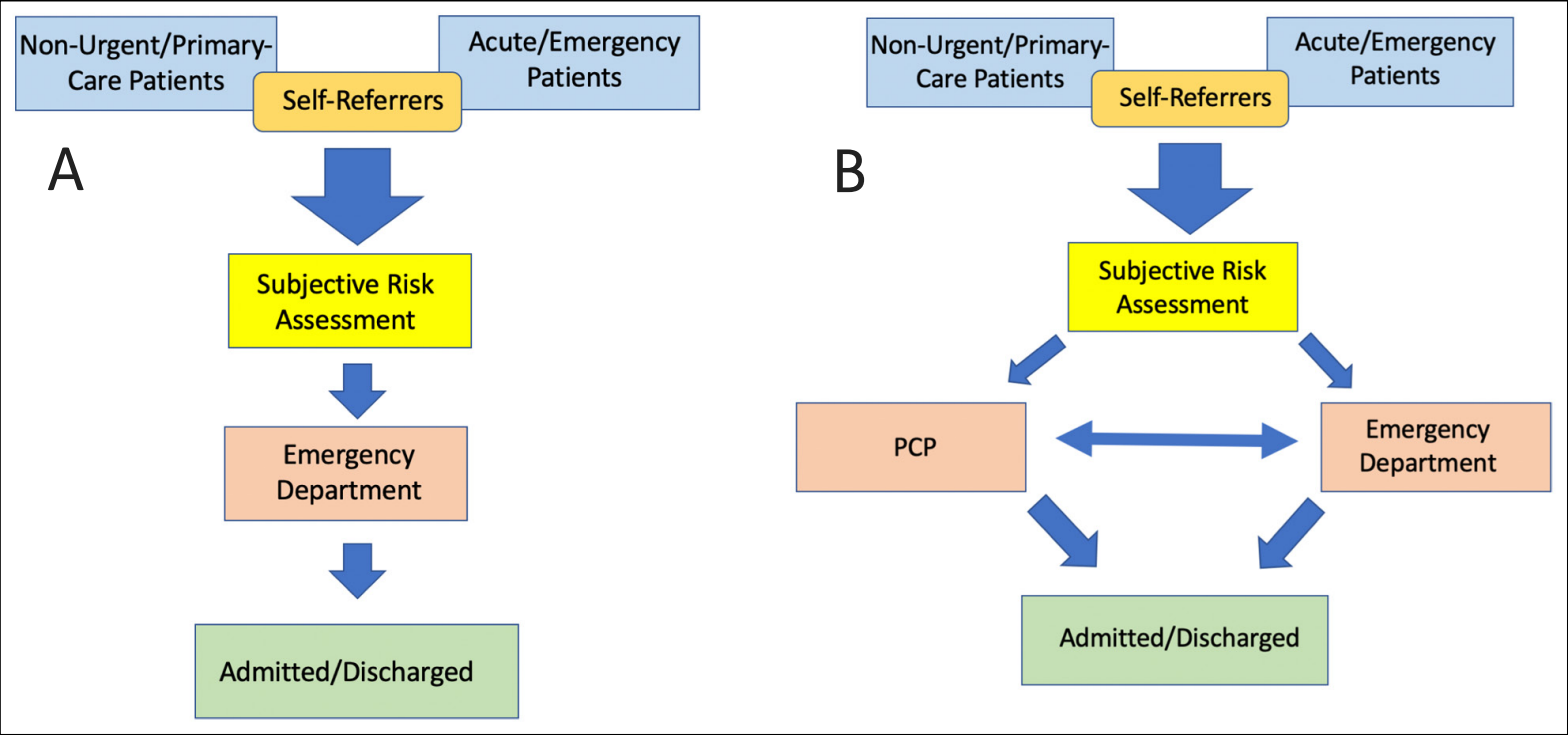
**(A)** Standard Patient Flow. **(B)** Patient Flow in PCP Streaming.

There was no objective triage process in Tübingen’s medical NA and no use of recognised triage systems [20]. The initial assessment of the patient was conducted subjectively by either a healthcare assistant or a nurse in a brief interaction as the patient reported to the registration window. Further examination, such as vital signs or review by a doctor, was conducted as deemed appropriate. Patients were streamed into the PCP pathway following this subjective risk-assessment. ***Figure 2*** depicts the patient flow pre- and post-intervention. For clarity, ‘triage’ will be used hereafter only to describe the use of a formal objective triage system.

A PCP treating patients will be one step on from the triage point. This is the case even in models whereby PCPs are the ‘triagers’. A triaging PCP who treats a patient is principally taking the patient to the step down-stream from triage. To place the singular PCP in our study within triage, treating ‘as they go’ and without parallel patient-flow would have caused bottlenecks, thus undermining the principle of triage. Such an approach can be achieved effectively with more resources, as in Basel, by using a ‘team-triage’ [16].

## Results

The pre-pilot-study data likely over-reported the number of self-presenters due to differing selection criteria to those adopted for the pilot. The pilot study proposed ‘primary care’ patients to be those of low urgency/acuity, self-referred, presenting for a new episode of care (i.e. not a planned return) and unlikely to be admitted [21]. This definition assumes a triage process, which this project lacked. Therefore, the ‘low urgency’ was established through the subjective assessment of the receiving NA staff.

After about 1 month it became apparent that by utilising an objective definition of a primary-care patient [21] the demand from primary care patients was not as high as first thought. It was agreed that the project would continue for 3 months and at which point the study PCP, the head of the medical NA and the clinical directors of the medical and general practice departments met to assess the preliminary results. It was agreed by all parties that the numbers were not conducive to continuing the pilot study.

The ‘Intervention Phase’ of the project ran for just over 3 months from 02.08.17 to 10.11.17 and during this time, which amounted to 66 working days, 88 patients were seen by the study PCP; 1.3 patients per 4-hour shift.

As to whether the low number of primary care patients reflected the demand and as to why it was unexpectedly low, is considered. The reflections here draw on the experiences of the study PCP and explore the structural and cultural barriers to this study. As such, this is an opinion-based article.

## Discussion

This project’s attempted PCP integration encountered several obstacles. Most obviously, demand for the service was not as high as first thought and we consider why there was an overestimation. The demand was likely there, but it was not feasible to capture the patients because of the main barriers to this project:

1. A decentralised emergency system
2. No standardised objective triage
3. A culture of professional tribalism

To assess the successes and challenges of an integrated care project objectively and dispassionately can be difficult. Identifying the relevant factors amongst the complex interwoven web of clinical systems, professional structures, workplace dynamics and information systems can be a challenge. The Rainbow Model of Integrated Care (RMIC) was developed to aide in this work and to provide clarity and structure to the study of integrated care [22]. It represents a very pragmatic and useful evolution with a focus on primary care integration. A taxonomy, a formal system to classify these otherwise abstract entities of integrated care, was also developed by Valentijn et al and further helps to structure the discussion [23,24].

**Figure 3:**
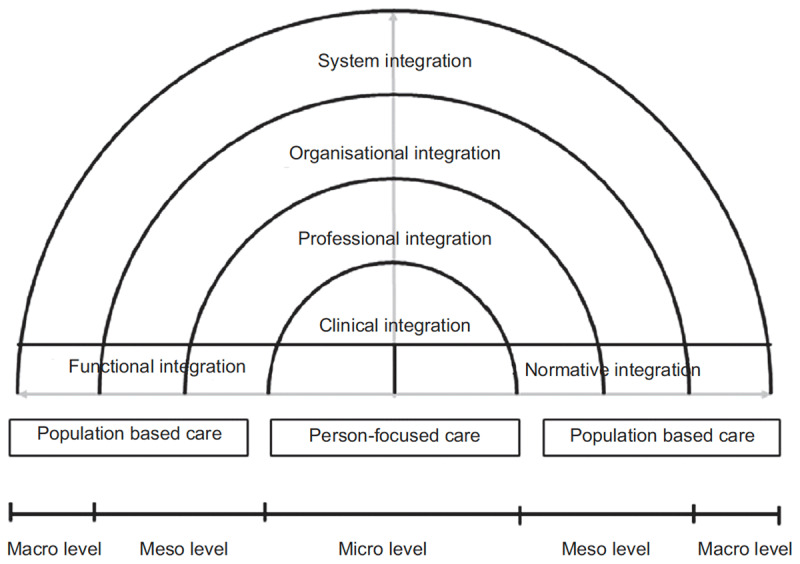
Conceptual framework for integrated care based on the integrative functions of primary care [22].

In the RMIC (***Figure 3***), there are 6 dimensions which, as per Hardy’s early assertion of vertical and horizontal integrated care [25], follow an x and y axis. Much like a patient’s path through ever larger healthcare entities, the vertical ‘y-axis’ ascends through the micro- (clinical integration), meso- (organisational and professional integration) and macro-level (system integration). Throughout, interlinking and connecting each tier of the vertical, are the horizontal dimensions of functional and normative integration.

Functional integration is perhaps best understood as the practical tools required for integration: the mechanisms which link data, financing and the management of human resources, strategic planning, information and quality improvement. Normative integration is the human element. It is the development and maintenance of a common frame of reference (i.e. shared mission, vision, values and culture) between organisations, professional groups and individuals [22].

The RMIC is used here as a tool to structure assessment of the myriad of human variables, which have influenced the success or otherwise of this integrated care project. The relevant dimensions of the RMIC form the headers below and the key attributes (italic font) of the RMIC dimensions, as defined in the taxonomy, are used to frame the discussion. The hope is to enable our experience to serve as a useful signpost for future PCP integration projects and, thus, to enable them to be truly integrative from the outset.

## A decentralised emergency system

### Clinical and Professional Integration

In the Anglo-American system, there is a distinct specialist field of emergency medicine and the specialist emergency practitioners operate within emergency departments and systems.

In Germany, healthcare is decentralised, different healthcare providers are responsible for hospital care and emergency medicine is not a distinct speciality. An emergency department is called a ‘Notaufnahme’ but this term is also used to describe what would be ‘acute admission units’ in other healthcare structures. The abbreviation ‘NA’ is used here to differentiate between the broader German entity and the narrower Anglo-American ‘emergency department’ or ‘ED’.

Tübingen’s main hospital complex is a manifestation of the above: It is composed of two healthcare providers (UKT and BG) and was developed piece by piece, spread over three separate sites, with different admitting sub-speciality NAs and clinical departments in often separate buildings. Despite perhaps the heightened need, Tübingen lacks a formal, centralised emergency department.

The lack of a centralised emergency department was the most palpable functional barrier to this integrative project. In Valentijn et al.’s taxonomy most of the key headers of clinical integration would be enabled or improved by the establishment of a centralised emergency department [23]. Not least, the centralising of key specialists and healthcare workers would enable *individual multidisciplinary care planning*. However, beyond simply co-locating personnel, a recognised, formalised and reproducible framework of emergency care - an emergency medicine specialty - could further augment care.

Acutely unwell or emergency patients are the patients who need a strong and successful inter-disciplinary team the most. The resuscitation team leader within an Anglo-American model emergency department is a senior emergency medicine doctor; they function like the conductor of an orchestra; they stand back and coordinate the resuscitation of the patient; they bring in the specialists at the appropriate junctures. As such, this is representative of how an emergency department, and the specialism of emergency medicine within it, can be the fulcrum at an organisational level and from which urgent or unplanned inter-disciplinary hospital care is initiated and coordinated.

Such a template at the micro-level would in turn impact the meso-level or beyond. It is difficult to imagine the growth of such a disparate hospital infrastructure occurring if emergency medicine was a recognised specialty with a seat at the table of infrastructure development.

An optimally coordinated emergency care system is a dominant *population need*. The embracing of emergency medicine, or at least the establishment of a centralised emergency department, would improve coordination of care for high-risk patients first and foremost (*case management*). In turn, those at lower risk and contributing to overcrowding could be fully captured and better managed through further clinical integration, such as that intended by this pilot study.

For non-urgent yet complex-needs patients the PCP could provide further coordination of inter-disciplinary care within the hospital walls and beyond (vertical and horizontal integration). The pilot could have aided in the provision of services with respect to medical, psychological and social aspects of health (*centrality of client* and *service characteristics)* and improved *continuity of care*. Outcomes worthy of future study. However, a central emergency department is the starting point; PCP-integration is the cart to the carthorse of a centralised ED.

## No standardised objective triage

### Functional Integration

The lack of a formalised triage system was a major hindrance to the project from the outset. The implementation of objective triage was resisted in planning because a formal triage process had been used previously but was stopped due to strains on the manning resources. However, a triage system is a recognised fundamental to emergency care, including in Germany [20,26], and is safer than a subjective risk-assessment [27].

An objective mechanism of initial assessment is a fundamental of a streaming pathway at the clinical micro-level and the first step in wider regional care coordination and resource management. An optimum triage system is one which is safe, efficient and easily reproducible. It should facilitate matching the available resources in a timely manner to the needs of the patient.

With respect to objective triage, there appeared to be a different cultural perception in which it was an optional extra rather than a fundamental. Tübingen was not alone in its needs-based approach to triage. A PCP integration workshop discussed this topic at the German Association of PCPs (DEGAM) Conference 2017 and the common experience of those attending was that their EDs did not absolutely apply triage in their departments or necessarily incorporate it rigorously within PCP-streaming design. Instead, centres had often relied on the subjective assessment of staff who first received the patient. Recently, and perhaps as a means to address this, new national funding incentives for NA in Germany were established to outline the future German emergency care system and define fundamentals of emergency care; these include objective triage [26,28].

#### Good governance and Available resources

Organisational integration requires *inter-organisational governance* sharing strategy, policy and responsibility. In emergency systems, a fundamental to this is a standardised and objective way to risk categorise the patients. A culture of triage and the objective stratification of the patient’s risk category is essential and relevant not just at the coal face of care but also at the meso- and macro-levels of resource allocation and joint care planning, such as in national trauma coordination [29]. Objective categories of risk are required to facilitate functional integration, thus enabling the matching of resources to demand. Further development of national trauma networks and an improved coordination between pre-hospital and hospital emergency services at a local level, plus coordinated regional and trans-regional emergency care, is already recognised in Germany [30]. The control centres coordinate with a focus on life-threatening cases. To fill the gap in the low-risk category there is a telephone triage algorithm for Germany currently under development with the expressed intention of alleviating ED overcrowding [31]. A cultural embracing of objective triage assessment tools at both the local and regional level is essential for coordinated emergency healthcare.

## A culture of professional tribalism

### Normative Integration

The dimension, which is less palpable and often overlooked, yet is essential for the success of an integration project, is Normative Integration. These are the human qualities, which are as crucial for the success of the project as they are difficult to measure. Even if all the functional qualities are present and optimised, the human factor is key.

***Table 3*** highlights the key features of Normative Integration. The following headers were found to be most relevant to our experience:

**Table 3:** The Key Features of Normative Integration (Adapted from ‘Taxonomy of 59 Key Features’) [23].


KEY FEATURES	DESCRIPTION

Collective attitude	Collective attitude within the collaboration towards open communication, sincerity and respect at operational, tactical and strategic levels.

Sense of urgency	Awareness regarding the need and purpose to collaborate at the operational, tactical and strategic levels.

Reliable behaviour	The extent to which the agreements and promises within the collaboration are fulfilled at operational, tactical and strategic levels

Conflict management	The ability to effectively manage interpersonal conflicts within the collaboration.

Visionary leadership	Leadership based on a personal vision that inspires and mobilizes people.

Shared vision	A collectively shared long-term vision within the collaboration at the operational, tactical and strategic levels.

Quality features of the informal collaboration	Effectiveness and efficiency of the informal collaboration at the operational, tactical and strategic levels (e.g. group dynamics and attention to the undercurrent).

Linking cultures	Linking cultures (e.g. values and norms) with different ideological values within the collaboration at the operational, tactical and strategic levels.

Reputation	Individual reputation of those people involved in the collaboration.

Transcending domain perceptions	The ability to transcend one’s own professional domain within the collaboration at the operational, tactical and strategic levels

Trust	The extent to which those involved in the collaboration at operational, tactical and strategic levels trust each other.


#### Transcending domain perceptions

The layout of the hospital complex did not help to encourage a joined-up approach. Specialties which needed to work together (e.g. in a trauma-resuscitation scenario) could be based in different buildings over the broad hospital site or even in different hospitals. The physical separation may have contributed to the atmosphere surrounding inter-disciplinary teamwork, which was observed to be strained and one of stark inter-sectoral borders and professional tribalism (i.e. sharply delineated departments). The delineation was also present between primary and secondary care (discussed below), but within hospital it was evident between hospital specialities, even within the compartmentalised emergency care provision of the NA. For example, the breadth of internal medicine at UKT is covered by nine separate administrative departments. The medical NA was staffed and administrated primarily by Internal Medicine Department 1 (IMD1: gastroenterology, hepatology, gastrointestinal oncology, infectious diseases and healthcare of the elderly). However, the scope of the care was such that it required further staffing by Internal Medicine Department 3 (IMD3: cardiology and angiology). Disease and ailments do not respect such constructed physiological boundaries and patients with multisystem disease or non-specific symptoms would often test these intradepartmental, intersectoral boundaries, particularly at busy periods or in high acuity patients. The relations between the departments directly impacted on the pilot study too; the study’s clinical room was suddenly reclaimed and re-purposed of by its owners (IMD3) soon after it became evident it was now in clinical use by IMD1.

The facets of Normative and Professional Integration would be powerfully enabled by a singular emergency department as well as a recognition of the emergency medicine specialty itself. Not least in the key features of *clinical leadership, inter-professional governance creating interdependence between professionals* and a *shared vision between professionals*. Many of these key features echo those qualities of communities of practice, which the formation of an emergency medicine speciality and centralised department would foster by bringing together the (hitherto distinct) specialities [32].

##### Quality features of the informal collaboration, Reputation and Trust

The frequency of self-referrers to the NA in our pilot study was below that expected because the expectation was falsely raised. Over the previous 6 months to the start of the study a preliminary data gather was conducted, which lacked a formal definition of a non-urgent patient and relied on a non-specific term of ‘Selbstvorsteller’ or ‘Self-presenter’. At the time, ‘self-presenters’ was topical and suspected by the department to be the main cause of overcrowding. In an overstretched department, a subjective appraisal of whether someone was a ‘self-presenter’ was particularly vulnerable to confirmation bias. There was a significant overestimate of the demand. The pilot study’s numbers fell far short of the 11–17 ‘self-presenters’ over the day in the preliminary data gather.

Implicit in the term ‘Self-presenter’ is the notion that the patient attends without a referral. However, this was not necessarily the case. Many did have referrals, but they were often for non-urgent care or, on occasion, were indeed urgent but deemed to be an overstatement of the problem. Finally, there were also those who fulfilled the descriptor of a ‘Self-presenter’ but who were nevertheless in the right place; acutely unwell patients could and would self- present without going through their PCP.

The points of apparent strain in a stressed system draw attention and criticism- the above being a symptom of this. Equally, if there is the perception that the PCPs are not doing their job well and that they are the openers of the floodgates, it is probable the value and respect attributed to the referral of a patient from a PCP is reduced.

Add to this that in Germany the same form is used for both same-day (urgent) and routine referrals, then there is scope for the urgent referral to be devalued through misuse (by the patients) or simple error (e.g. misreading).

The *type* of PCP seemed to affect the worth of the referral too. PCPs in Germany can be of two categories. Either a community-based internal medicine physician, who completes their training in hospital, or a ‘General-Practitioner(GP)’ PCP whose training comprises of hospital rotations and primary care placements. There was the impression that a referral from the GP-PCP was often held in lower regard than the Internal Physician PCP. A bias, if present, likely originating from the perception of the lesser rigor and governance in the GP-route of training. Much work has been done to formalise and standardise the GP-route of training in Germany with vocationally specific rotations [33,34]. In the region of Baden-Württemberg, a formalised PCP postgraduate programme has been running since 2015 and how this is changing perceptions is the focus of a current prospective study.

However, PCPs contribute to the problem too. Despite a system of community-based specialists and generalists the patient with complex needs, and especially impaired mobility, may suffer difficulty in accessing the community practices or securing home visits. Often, they are referred to hospital care for non-specific reasons such as ‘general deterioration’ perhaps following only a telephone consultation. The hospital physicians can perceive this as a misuse of the system. If we consider too that social reasons, shortcomings in elderly care and risk-liability often underly PCP-referrals, then it can further antagonise. Even when the system is correctly utilised it can engender confirmation bias: PCPs often need to have the NA rule out an urgent differential diagnosis rather than the lower-acuity suspected diagnosis. The ‘rule out’ diagnosis is thus ruled out, but the ‘quality’ of the referral is perceived negatively. In anticipation, the PCP may seek to avoid any ‘sell’ of the patient to their hospital colleagues and opt to send the patient unannounced (i.e. no telephone handover), albeit with the brief referral form. This also antagonises.

Such behaviours are symptoms of structural deficits in healthcare, which can actively erode *trust* and *informal collaboration* between the NA and primary care. Altogether, the PCP referral appeared at times not to be respected and patients who had been referred were on occasion, nevertheless, streamed as primary-care patients.

##### Collective Attitude, Linking Cultures and Trust

The role of PCPs as ‘gatekeepers’ and their perception amongst secondary care colleagues is also relevant to their success as interdisciplinary coordinators. There has been some interest recently in the perceived “[PCP] bashing” prevalent in the UK [35,36]. The study PCP’s experience of how PCPs and their referrals were perceived by their secondary care colleagues echoed such sentiments. There are few inter-sectoral borders as stark as that between primary and secondary care. There are initiatives in both the UK and Germany trying to blur this boundary for a smooth patient journey. However, if the PCPs, the gatekeepers of the healthcare system, are perceived as a contributor to the strain on resources by secondary care practitioners, rather than useful allies, this causes an atmosphere of mistrust and negativity. There is also a risk of this negativity perpetuating itself. Budding doctors’ perception of primary care can quickly become negative if they consistently hear disparaging comments, which risks propagating the cycle [35].

Any change from the status quo presents challenges and resistance. Key to overcoming these are *visionary leaders* and stakeholders who have respect and trust for each other. The normative dimension transcended each of the barriers discussed and was the dimension which, on reflection, most influenced the success or otherwise of this study.

## Were these integrated care project experiences typical?

The lessons learnt from this pilot study appear to align with the insights of other studies. Vantijn et al. identified in their assessment of 69 integrated care projects in the Netherlands the factors affecting the evolution of collaborative processes and which inferred success or otherwise [37]. They established three subgroup categories for each of the projects depending on scores relevant to the RMIC. Perhaps unsurprisingly, the projects with United Integration Perspectives, which scored above average in system, organisation and professional integration were perceived as most effective. To the contrary, the projects with ‘Disunited Integration Perspectives’ were perceived to be the least effective. In a parallel publication using the same Dutch integrated care projects, a better understanding of the collaboration processes and their relationship to the perceived success of the partnership was sought [38]. The outcome: “Partnerships that were more positive about mutual gains and process management at baseline had a significantly higher level of perceived success,” and “partnerships that demonstrated an increase in relationship during the collaboration process also had higher levels of perceived success.” The authors reasonably contend that such relationship dynamics, or trust-based governance mechanisms, are of more importance than organisational dynamics. Or put simply, normative integration trumped functional integration.

The other similar German pilot projects, which were already up and running in Hamburg and Hannover, were successful and displayed such attributes. Firstly, each (and especially Hamburg) benefitted from an already established collaboration between the PCPs and their secondary care colleagues on the hospital site. Plus, each had a centralised emergency department, which fundamentally channelled enough primary care patients. Importantly too, their contexts benefitted from already established elements of functional integration (i.e. data, financing and strategic planning) and normative integration. Their working relationships were already formed through parallel projects (e.g. Hamburg’s on-site PCP clinic provided on-call cover for secondary care patients in a nearby hospital building) and the relationships between collaborators appeared to increase as the project matured. Also apparent were the palpable mutual gains for each collaborator. In the Tübingen study this was less apparent. The primary motivator for the overcrowded emergency/acute care provider was to alleviate the perceived burden on the system. However, the identity of the source of the problem changed with scrutiny, there were no longer mutual gains and, in the end, the barriers to the originally intended collaboration were too inhibitory.

## Conclusion

Traditional healthcare structures are at risk of becoming anachronisms in a patient-centred culture in which the patient is frustrated of having limited pre-defined entry-points imposed. The patient is no longer the passive recipient but the informed consumer. PCP integration is not a tool to transform systems into demand-driven entities but rather its relevance will increase in systems more responsive to the patient’s needs, including those who find it difficult to access healthcare.

PCPs as ‘interventions’ in unplanned care are commonly the precipitation of attempts at integration at a structural and organizational level. However, the outcome does not typically manifest as integrated care. This may be more a consequence of misinterpreting what integrated care is rather than the suitability of the PCP in providing it. Indeed, the role of a PCP in society and the organisational knowledge they confer could aid the transition between primary and secondary care and between hospital and community care; vertical and horizontal integration.

There were barriers to care which impacted the pilot study. Some of which were organizational and structural, others were cultural. A lack of normative integration was a key feature of each barrier to this project. To the contrary, normative integration was a common factor in successful integration studies. It is hoped the lessons learnt and the framing of our experiences within the RMIC model, will help guide other PCP-integration projects to a clearer understanding of clinical integration and help in the early identification of the key normative elements to enable a successful project.

## Limitations

Although an opinion piece, the opinions drawn relate to the authors’ experiences of studies and projects from departments of typically university hospitals. Such a ‘self-selected group’ is not a representative spread of experiences from the different types of hospitals in Germany. Many integration projects have been successfully implemented in non-university hospitals (as was also evident at the DEGAM PCP/Notaufnahme Integration Workshop 2017) and their experiences and data not necessarily published. Of relevance, the study PCP has also worked recently in a non-university, regional, central Notaufnahme in Germany, which has helped shape the opinions put forward.
